# Protective Role of Coxsackie-Adenovirus Receptor in the Pathogenesis of Inflammatory Bowel Diseases

**DOI:** 10.1155/2018/7207268

**Published:** 2018-08-09

**Authors:** Xiong Chen, Rui Liu, Xiaoming Liu, Canxia Xu, Xiaoyan Wang

**Affiliations:** Department of Gastroenterology, The Third Xiangya Hospital of Central South University, Changsha City, Hunan Province, China

## Abstract

**Aim:**

To investigate the role of Coxsackie-adenovirus receptor (CAR) in inflammatory bowel disease (IBD).

**Background:**

CAR, a type I transmembrane protein with functions in virus attachment, has been shown to be associated with epithelial tight junctions (TJs) and mediates cell adhesion, implying its potential roles in the pathogenesis of IBD.

**Methods and Materials:**

To determine the effect of CAR in IBD using QPCR and Western blotting to determine the expression of CAD in TNF-*α* induced NCM460 and SW480 cells and IBD tissues compared to control groups. Furthermore, TJs dysregulation, FITC-Dextran permeability assay, qRT-PCR, Western blot, and IF assessed the permeability in CAR overexpressed cells treated with TNF-*α*. HE, qRT-PCR, Western blot, and IHC assay were used to assess the CAR overexpressed cells whether they have the effect to cure DSS induced ulcerative colitis rat model* in vivo*.

**Result:**

We found CAR levels in human colon cell lines are significantly downregulated under the treatment of tumor necrosis factor-alpha (TNF-*α*). Furthermore, overexpression of CAR markedly prevented TNF-*α* induced inflammatory response, TJs dysregulation, and permeability disruption (FITC-Dextran permeability assay) in cells. Consistent with these findings* in vitro*, we found that CAR overexpression could suppress gut inflammation, attenuate the downregulation of TJ protein ZO-1 and Occludin, and limit the induction of barrier permeability in a DSS induced ulcerative colitis rat model* in vivo*. Together, our findings strongly suggest that CAR could protect tight junctions and has an anti-inflammatory effect during the pathogenesis of IBD. Thus CAR may serve as a therapeutic target for the diagnosis and treatment of IBD.

## 1. Introduction

Inflammatory bowel disease (IBD) is chronic inflammatory disorder of the gastrointestinal (GI) tract and has become public health concern due to its increasing incidence worldwide during the past few years [1]. IBD are clinically characterized by symptoms such as abdominal pain, bloody diarrhea, weight loss, and fatigue [2]. Based on clinical and pathologic pattern, IBD can be divided into Crohn's disease (CD) which affects GI track in a discontinuous and transmural manner and ulcerative colitis (UC), which affects restrictedly the surface of mucosa of the colon and rectum [3, 4]. The etiology of IBD remains unclear, and it is presently recognized that pathogenesis of IBD is contributed by multidimensional factors that involve environmental, genetic, microbial, and immune components [5, 6]. Dysregulation of intestinal immune response, which can be initiated by upregulated host defense reaction of intestinal epithelium to bacteria, promotes perpetuation of inflammatory response in IBD [7]. Upregulation of inflammatory cytokines and activation of inflammation signaling pathways are critical contributing factors to pathogenesis of IBD [8]. Besides, compromise of gut epithelial barrier is another important characteristic of IBD, and dysregulation of tight junction (TJ) proteins is key mechanism for disruption of paracellular permeability during pathogenesis of IBD [9].

Although multiple therapeutic options such as treatment with steroids, immunomodulators, and antibodies are available for the treatment of IBD, clinical applications of the present treatments are limited due to various adverse effects including infections and malignancies [10–12]. Other options such as nutritional therapy are limited on efficacy [13]. Thus, better understanding of the mechanisms of disease pathology is in urgent need in order to identify novel drug targets for the treatment of IBD. Tight junctions which form the apical unit of GI track are the predominantly rate-limiting factor in paracellular passage of intestinal barrier [14]. Transmembrane proteins such as occludin and peripheral membrane proteins such as zona occludens (ZO-1, ZO-2, and ZO-3) are major components for building up tight junctions [9]. Importantly, a transmembrane protein Coxsackie-adenovirus receptor (CAR) has been reported to play critical role in maintaining barrier function of TJs [15]. CAR belongs to the CTX subfamily of the immunoglobulin superfamily, and it is originally known to be the primary attachment protein for viruses to enter the cells [16]. Although the cellular function of CAR is largely unknown, recent studies suggested that CAR mediates cell-cell adhesion through its association with epithelial TJs [17], suggesting the potential role of CAR in regulating permeability of gut barriers—an important indicator of IBD pathogenesis.

In the present study, we aimed to explore the role of CAR in the development of IBD. We found that CAR exerts significant beneficial effect through suppressing inflammatory signaling activation, inhibiting inflammatory cytokines production, and upregulating the expression of TJs, supporting that CAR might act as a protective factor during the pathogenesis of IBD. These findings may shed light on a novel therapeutic target for the future diagnosis and treatment IBD of CAR.

## 2. Material and Methods

### 2.1. Cell Culture & Treatment

Human colon mucosal epithelial cell line (NCM460), human colon cancer cell line (SW480), and human epithelial colorectal adenocarcinoma cell line (Caco2) were purchased from Auragene cell bank (Changsha, Hunan, China). Cells were maintained in humidified cell incubator (5% CO_2_, 37°C) with respective cell culture medium (RPMI 1640 medium (Gibco, Carlsbad, CA, USA) supplemented with 10% fetal bovine serum (FBS, Gibco) and 100 U/ml penicillin-streptomycin). Cells were split into 6-well plates in order to reach 50% confluence at the time of treatment or transfection. For CAR overexpression, CAR gene was cloned into pEGFP-N1 vector via NheI and BamHI cloning sites, sequence was verified, and the recombinant plasmid was transfected into cells using Lipofectamine 6000 reagent according to manufacturer's instruction. 

### 2.2. Animal and DSS Induced IBD Model

BALB/c mice were used to establish DSS induced IBD model. Briefly, male BALB/c mice aged 4–6 weeks (bodyweight 23–25 g) were randomly divided into 3 groups (8 mice per group). All animals were housed in 25°C environment with a 12-hour light and dark cycle. For the first 7 days of experiment, control group was maintained with free access to regular drinking water, while IBD group and CAR-IBD group were given free access to drinking water containing 5% DSS. Starting from day 8, all groups were given free access to regular drinking water. On day 9, CAR-IBD group was infected with CAR overexpression adenovirus through rectal perfusion. During the experimental process, observation and records were made on the bodyweight, food intake, activity, and fecal characteristics of all treatment groups. On the 17^th^ day of experimental scheme, mice were sacrificed and colon tissues were collected for further analysis of gene expression and histological studies. To evaluate the effectiveness of IBD model as well as the influence of CAR overexpression on IBD development, disease activity index (DAI) scores were generated based on the following criteria: no weight loss= score 0, bodyweight loss <5% = score 1, bodyweight loss between 5%–10% = score 2, bodyweight loss between 10%–15% = score 3, and bodyweight loss between >15% = score 4. Fecal characteristics were also scored ranging from 0 to 4 based on the texture and extent of blood content.

### 2.3. Immunocytochemistry

After treatment/transfection, cells were fixed by paraformaldehyde and washed with PBS before being incubated with primary antibody (Anti-ZO-1 antibody, Proteintech, # 21773-1-AP, 1 : 100, Wuhan, China). Fluorescence conjugated secondary antibody (goat anti-rabbit IgG-CY3, Auragene, #SA004, Changsha, China) was added onto cell slides and incubated for 30 minutes at room temperature. Cells were then washed by PBS for 3 times (3 minutes each time), stained with 4′,6-diamidino-2-phenylindole (DAPI), and preserved in antifade mounting medium (Auragene, #P0371H, Changsha, China) for subsequent microscopic observation.

### 2.4. Quantitative Real-Time PCR (qRT-PCR)

Total RNA of cell lines and colon tissue samples were extracted according to instructions of Trizol kit (Dongsheng Bio. # R1022, Guangzhou, China). mRNA was reverse transcribed into cDNA with reverse transcription kit (ThermoFisher, # K1622, USA) according to the manufacturer's instruction. Gene expression level was determined by quantitative real-time PCR using SYBR Green qRT-PCR Mix (Dongsheng Bio. # P2092, Guangzhou, China) with ABI 7300 real-time PCR system. Primer information for qRT-PCR is as follows: CAR-Sense 5′-TGTGCGGAGTAGTGGATT-3′, CAR-Antisense 5′-ATGGCAGATAGGCAGTTT-3′, *β*-actin-Sense 5′-AGGGGCCGGACTCGTCATACT-3′, *β*-actin -Antisense 5′-GGCGGCACCACCATGTACCCT-3′, IL-21-Sense 5′-TGAATGACTTGGTCCCTG-3′, IL-21-Antisense 5′-GTGTTTCTGTCTTCTCCCT-3′, IL-23-Sense 5′-GGGACACATGGATCTAAGAG-3′, IL-23-Antisense 5′-CGATCCTAGCAGCTTCTCAT-3′, ZO-1-Sense 5′-GACAGCTACAGGAAAATGACC-3′, ZO-1-Antisense 5′-CCTTCTAATTGTAATTTTTGCA-3′, Occludin-Sense 5′-CGGTACAGCAGCAATGGTAA-3′, and Occludin-Antisense 5′-CTCCCCACCTGTCGTGTAGT-3′. All qRT-PCR reactions were performed in triplicate, and *β*-actin gene was used as endogenous control.

### 2.5. Western-Blot Analysis

Cells were harvested (tissue samples were homogenized) in RIPA lysis buffer (Auragene, # P002A, Changsha, China) containing proteinase inhibitors. After 20 min incubation on ice, protein mixture was extracted by centrifuging samples at 13000 rpm for 20 min. BCA assay was used to determine protein concentration before samples were diluted into 2 mg/mL with sodium dodecyl sulfate-loading buffer (Auragene, # P003B, Changsha, China). For immunoblot assay, equal amounts of protein from each sample were electrophoresed on a 10% sulfate-polyacrylamide gel and transferred onto nitrocellulose membrane. 5% milk blocked membranes were then incubated with primary antibody in 3% BSA-TBST overnight at 4°C, washed by TBST, and incubated with secondary antibody. After a second wash, results were detected using AuraECL chemiluminescence kit (Auragene, # P001WB-1, Changsha, China). Antibody information is as follows: Anti-ZO-1 antibody (Abcam, # Ab96587, 1 : 2000, UK), Anti-Occludin antibody (Abcam, # Ab222691, 1 : 500, UK), Anti-CAR antibody (Abcam, # Ab91605, 1 : 100000, UK), Anti-P65 antibody (Abcam, # Ab32536, 1 : 8000, UK), goat anti-rabbit IgG-HRP (Auragene, # SA009, 1 : 15000, Changsha, China), and goat anti-mouse IgG-HRP (Auragene, # SA001, 1 : 15000, Changsha, China).

### 2.6. Immunohistochemical Staining

Colon samples were collected, fixed with 10% formalin, and embedded into paraffin blocks. Antigen retrieval was performed in boiled Sodium Citrate buffer (Auragene, # P019IH, Changsha, China). Dewaxed tissue sections were rehydrated and treated with 3% hydrogen peroxide. For immunohistochemical staining, nonimmunized goat serum was used to block the tissues, and sections were then incubated with ZO-1 antibody (Proteintech, # 21773-1-AP, 1 : 200, Wuhan, China ) overnight at 4°C, were washed with phosphate buffered saline, and were incubated with horseradish peroxidase- (HRP-) conjugated secondary antibody (Auragene, # SA009, Changsha, China). Signal was detected using DAB staining (Auragene, # P013IH, Changsha, China).

### 2.7. FITC-Dextran Permeability Assay

For* in vivo* permeability evaluation, 200 *μ*L FITC-Dextran (25 mg/mL, 40000 Da, Sigma, Cat. #53379, USA) was given to mice through gavage, 30 minutes later, blood was collected and plasma sample was preserved in light protected tubes, and further photometric analysis of FITC-Dextran concentration was detected using fluorescence microplate reader. For cell permeability evaluation, cells were seeded onto insert chambers which contain semipermeable membrane at the bottom, the chambers were then inserted into individual 12-well culture plates, after formation of cell monolayer, cells were treated with TNF*α* and/or CAR overexpression, and FITC-Dextran was then added to the inserted chamber in contact with the cell monolayer. The concentration of FITC-Dextran in the lower compartment of the culture well was measured to evaluate the permeability of the cell monolayer.

#### 2.7.1. Transepithelial Electric Resistance (TEER) Measurement

Cell monolayers were established on the polyester membrane of transwell inserts. After being treated by TNF-*α* and CAR transfection, we assessed the TEER using Millicell-ERS voltohmmeter (Millipore, Bedford, MA, USA). Briefly, electrodes were prewarmed to 37°C in HBSS and balanced for 20 minutes. Cells were washed by prewarmed HBSS and were incubated in fresh HBSS. Resistance value was measured for each well with a blank well used as control. TEER (Ω·cm^2^)= (resistance of cell – resistance of blank) x membrane area.

### 2.8. Statistical Analysis

Comparisons of difference between 2 groups for protein levels, mRNA levels, fluorescence intensity, and animal phenotype indicators were performed with Student's 2-tailed* t*-test, and for more than two groups Analysis of Variance (AVONA) was used. Data were analyzed using SPSS 17.0 statistical software, represented as mean ± s.e.m. P<0.05 was considered as of statistical significance.

## 3. Results

### 3.1. Expression of CAR Is Suppressed by TNF-*α* Treatment in Human Colon Cell Lines

**Figure 1:**
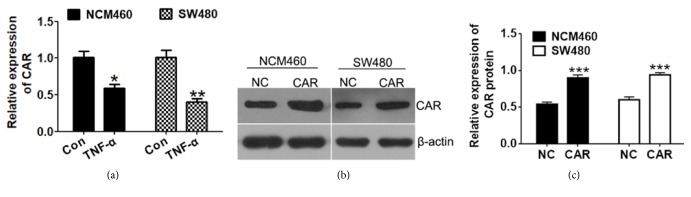
*CAR expression is inhibited by inflammatory factor TNF-α treatment in human colon cell lines.* (a) qRT-PCR detection of CAR mRNA levels in human colon cell lines upon TNF-*α* treatment. Human colon mucosal epithelium cell line NCM460 and human colon cancer cell line SW480 were treated with control or TNF-*α* (10 ng/ml) for 24 hours before cells were harvested for qRT-PCR assay. (b) Western-blot detection of CAR protein level after CAR overexpression. NCM460 and SW480 were transfected with control vector or pEGFP-N1-CAR overexpression plasmid, 48 hours after transfection, cells were harvested for protein extraction, and CAR protein level was determined using antibody that specifically recognizes CAR. (c) Quantitative analysis of CAR protein levels (calibrated with *β*-actin levels of each sample) in (b). ^*∗*^P < 0.05, ^*∗∗*^P < 0.01, and ^*∗∗∗*^P < 0.001, and data represent mean ± s.e.m.

We examined the expression of CAR in human colon (cancer) cells exposed on TNF-*α*, a well-known factor to induce inflammation. As shown in Figure 1(a), treatment of TNF-*α* significantly suppressed CAR mRNA levels in both NCM460 and SW480 cells (P<0.05), suggesting CAR expression is negatively correlated with inflammation in human colon cells. In order to study the function of CAR in colon cell lines, we then constructed CAR overexpression plasmid pEGFP-N1-CAR and transfected it into NCM460 and SW480 cells, and Western-blot method has successfully detected significant increased CAR protein level in the overexpression group comparing to control (P<0.05; Figures 1(b) and 1(c)).

### 3.2. CAR Overexpression Prevents TNF-*α* Induced Inflammation in Human Colon Cells

**Figure 2:**
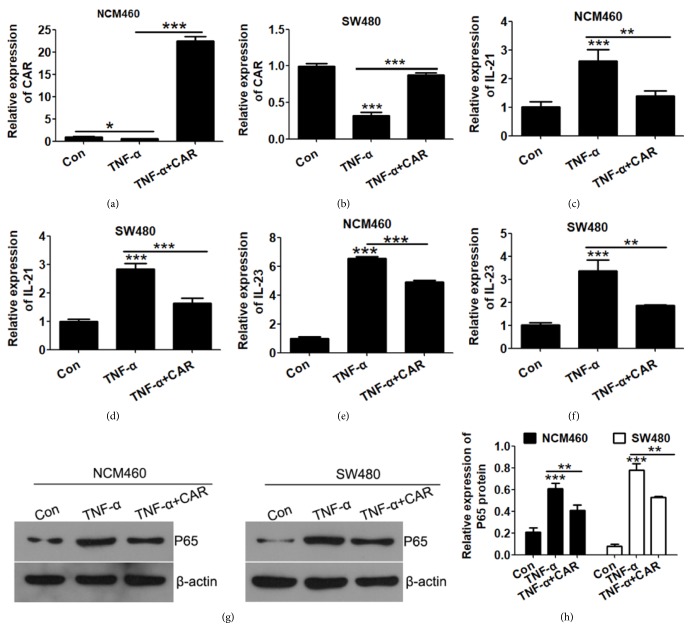
*CAR overexpression suppresses TNF-α induced inflammation in human colon cell lines.* NCM460 and SW480 cells were transfected with CAR overexpression plasmid and treated with control or TNF-*α*; cells were then harvested for qPCR detection and Western-blot assay. (a & b) CAR mRNA level were detected by qRT-PCR in CAR overexpression human colon cells NCM460 (a) and SW480 (b). (c–f) mRNA levels of inflammatory genes IL-21 (c & d) and IL-23 (e & f) expression upon CAR overexpression. (g) Western-blot examination of protein levels of inflammatory gene P65 and cell adhesive gene upon TNF-*α* and CAR overexpression in human colon cells lines. *β*-Actin levels of each sample were used as endogenous loading control. (h) Quantitative analysis of P65 protein levels (calibrated with *β*-actin levels of each sample) in (g). All data represent at least 3 independent experiments. ^*∗*^P < 0.05, ^*∗∗*^P < 0.01, and ^*∗∗∗*^P < 0.001; data represent mean ± s.e.m.

Since CAR expression was greatly downregulated upon TNF-*α* treatment in human colon cell lines, we next asked the question whether replenishment of CAR expression would counteract the effects of TNF-*α*. To test this hypothesis, we overexpressed CAR in NCM460 and SW480 human colon cell lines and treated cell with TNF-*α* (P<0.05; Figures 2(a) and 2(b)). TNF-*α* treatment alone greatly induced inflammatory genes IL-21 and IL-23 expression, while CAR overexpression significantly attenuated this induction in both cell lines (P<0.05; Figures 2(c), 2(d), 2(e), and 2(f)). Consistently, protein levels of inflammation marker NF*κ*B was induced by TNF-*α* and suppressed by CAR overexpression (P<0.05; Figures 2(g) and 2(h)). These results suggest that CAR prevents inflammation and helps maintain cell junction in human colon cell lines.

### 3.3. CAR Overexpression Prevents TNF-*α* Induced Upregulation of Cell Permeation in Human Colon Cells

**Figure 3:**
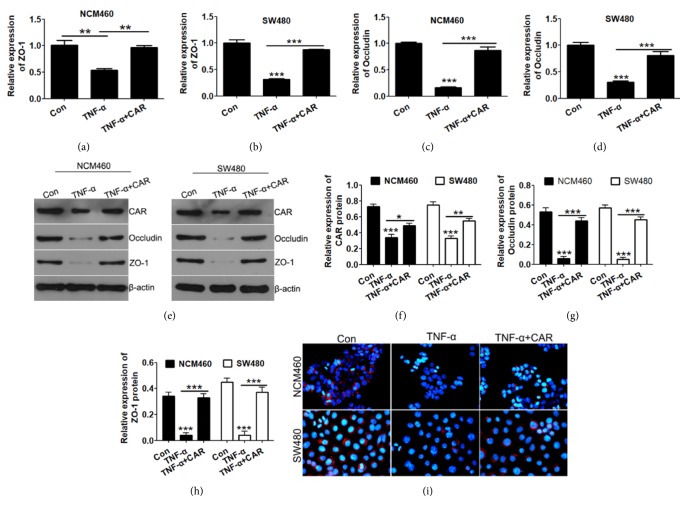
*CAR overexpression protects human colon cells from TNF-α induced TJ disruption*. NCM460 and SW480 cells were transfected with CAR overexpression plasmid and treated with control or TNF-*α*, and cells were then harvested for qRT-PCR, Western blot, and IF assay detection. (a–d) mRNA levels of cell adhesive marker gene expression ZO-1 (a & b) and Occludin (c & d) upon CAR overexpression detected by qRT-PCR assay. (e) Western-blot examination of protein levels of cell adhesive gene (ZO-1, Occludin, and CAR) upon TNF-*α* and CAR overexpression in human colon cells lines. *β*-Actin levels of each sample were used as endogenous loading control. (f–h) Quantitative analysis of CAR, ZO-1, and Occludin protein levels (calibrated with *β*-actin levels of each sample) in (e). (i) Immunocytochemistry staining detection of ZO-1 protein level after CAR overexpression explored on TNF-*α* of NCM460 and SW480 cells by IF. ^*∗*^P < 0.05, ^*∗∗*^P < 0.01, and ^*∗∗∗*^P < 0.001; data represent mean ± s.e.m.

**Figure 4:**
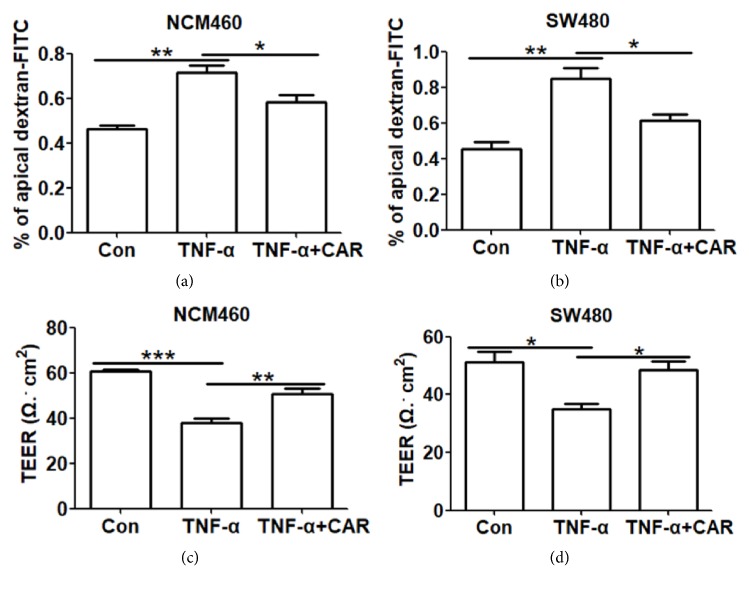
*Overexpression of CAR attenuates on TNF-α induced cell permeabilization in human colon cell lines*. The effect of CAR overexpression on NCM460 and SW480 monolayer barrier function. (a & b) FITC-Dextran cell permeability assay evaluation of human colon cell permeability upon CAR overexpression. Monolayers of NCM460 (a) and SW480 (b) cells were plated on upper-chamber of 12-well plates, after CAR overexpression and TNF-*α* induction, FITC-Dextran reagent was added to the upper-chamber, and, 30 min later, the concentration of FITC-Dextran in the lower compartment of 12-well plate was measured to determine the permeability of cell monolayers. (c & d) Effect of CAR on TEER of human colon cell lines. NCM460 (c) and SW480 (d) cells were transfected with CAR overexpression plasmid or control plasmid and treated with control or TNF-*α*, and TEER was measured to determine the barrier integrity of cells. All data represent at least 3 independent experiments. ^*∗*^P < 0.05, ^*∗∗*^P < 0.01, and ^*∗∗∗*^P < 0.001; data represent mean ± s.e.m.

We further confirmed the protective role of CAR by cell permeability evaluation. Treating cells alone with TNF-*α* downregulated the mRNA levels of tight junction gene ZO-1 and Occludin, and overexpression of CAR significantly reversed the suppression of ZO-1 and Occludin (P<0.05; Figures 3(a), 3(b), 3(c), and 3(d)). Further, protein levels of epithelial marker ZO-1 and Occludin were inhibited by TNF-*α* and upregulated upon CAR overexpression (P<0.05; Figures 3(e), 3(f), and 3(g)). Consistently, immunocytochemistry showed significantly decreased ZO-1 staining upon TNF-*α* treatment, while CAR overexpression greatly prevented this effect in both cell lines (P<0.05; Figure 3(h)). Finally, as shown in Figures 4(a) and 4(b), CAR overexpression largely decreased FITC-Dextran penetration through cells induced by TNF-*α*. We next measured the TEER of cells according to method described before [18]; consistently, CAR significantly increased TEER of cells upon TNF-*α* treatment in both NCM460 and SW480 cells (P<0.05; Figures 4(c) and 4(d)). Thus, CAR prevents barrier dysfunction induced by TNF-*α* on both functional and structural levels.

### 3.4. Overexpression of CAR Protects Mice from DSS Induced IBD

**Figure 5:**
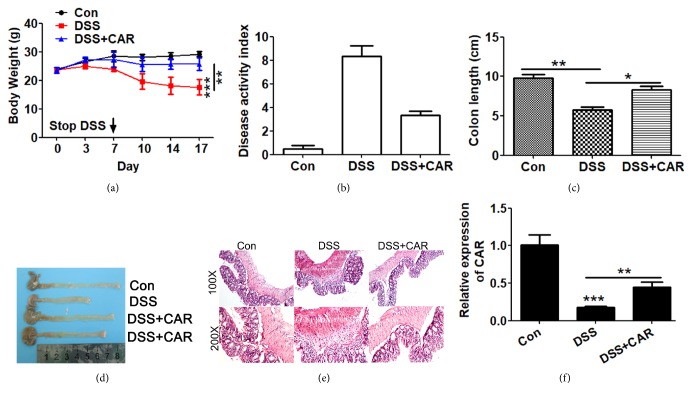
*Effect of CAR overexpression on DSS induced IBD development.* (a) Bodyweight of different treatment groups during establishment of DSS induced IBD rat model. Day 0–day 7 indicates time frame for control/DSS containing water treatment. (b) Disease activity index (DAI) of different treatment groups. DAI was calculated as the sum of bodyweight loss score, fecal texture score, and fecal blood content score collected at the end of the study. (c & d) Colon length of different treatment groups at the end of the study. (e) H&E staining of colon tissue. (f) Expression of CAR was determined by qPCR. Colon tissue samples were collected after animals were treated with control or DSS/DSS+CAR, mRNA was isolated, and genes were detected by specific primers. ^*∗*^P < 0.05, ^*∗∗*^P < 0.01, and ^*∗∗∗*^P < 0.001; data represent mean ± s.e.m. from 3 independent experiments with sample size = 3 for each group.

In order to test the physiological role of CAR on regulating colon barrier function, a mouse model of DSS induced colitis was established. Briefly, mice were treated with DSS to induce colonic inflammation and colitis, CAR was overexpressed through adenoviral infection, and, during this process, phenotype of mice and gene expression of colon tissues were compared among different treatment groups to determine the effect of CAR. As shown in Figure 5, significant bodyweight loss was observed in DSS treatment group comparing to control group, while the overexpression of CAR ameliorated the weight loss (Figure 5(a)). During the experimental process, bodyweight and fecal characteristics were calculated as disease activity index (DAI) to evaluate the extent of disease development. As shown in Figure 3(c), DSS dramatically induced disease activity, and CAR overexpression exerted significant protective effect.

At the same time, DSS administration greatly decreased colon length and this phenotype was rescued by CAR overexpression (P<0.05; Figures 5(c) and 5(d)). Further histological evaluation of colon tissue by H&E staining indicated that DSS treatment resulted in neutrophilic infiltration, scattered villus, and desquamation, while CAR overexpression greatly rescued the colon damage induced by DSS (P<0.05; Figure 5(e)). Finally, in vivo assay, we found CAR was decreased in DSS group, and CAR overexpression dramatically reversed DSS induced CAR downregulated (P<0.05; Figure 5(f)).

### 3.5. The Effect of CAR on Inflammatory and Tight Junction Gene Expression of Colon Tissues

**Figure 6:**
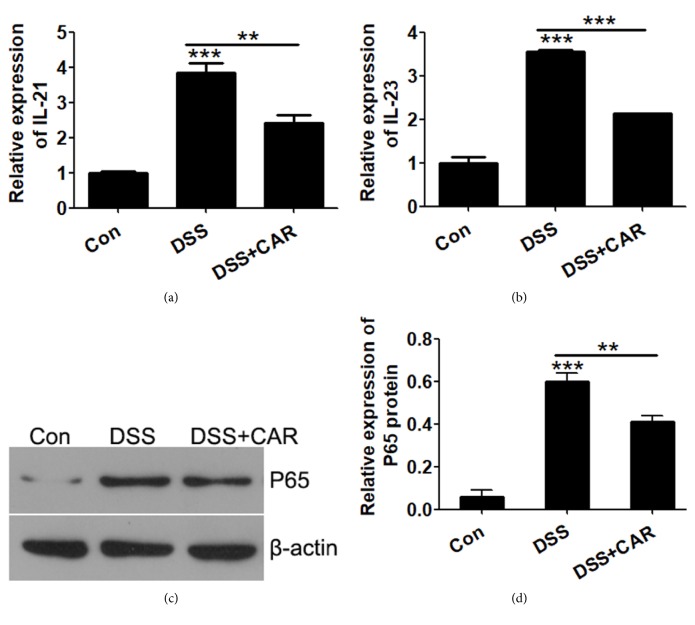
*Effects of CAR overexpression on DSS induced inflammation in vivo.* (a & b) Expressions of inflammatory genes IL-21 and IL-23 were determined by qPCR. Colon tissue samples were collected after animals were treated with control or DSS/DSS+CAR, mRNA was isolated, and genes were detected by specific primers. (c) Protein levels of tight junction gene P65 were determined by Western blot using specific antibodies, and *β*-actin levels were used as endogenous loading control. (d) Quantitative analysis of P65 protein levels (calibrated with *β*-actin levels of each sample) in (c). ^*∗*^P < 0.05, ^*∗∗*^P < 0.01, and ^*∗∗∗*^P < 0.001; data represent mean ± s.e.m. from 3 independent experiments with sample size = 3 for each group.

To further explore the mechanisms of CAR on preventing colon damage, we examined the gene expression of colons that were treated with DSS or DSS+CAR. Inflammation is one of the major factors during the progression of IBD, and, as shown in Figures 6(a) and 6(b), DSS administration significantly enhanced gene expression of inflammatory cytokines IL-21 and IL-23, while overexpression of CAR greatly suppressed IL-21 and IL-23 levels of both. Moreover, NF*κ*B signaling pathway was significantly induced by DSS in colon tissues, and CAR overexpression suppressed the activation of NF*κ*B (Figures 6(c) and 6(d)).

**Figure 7:**
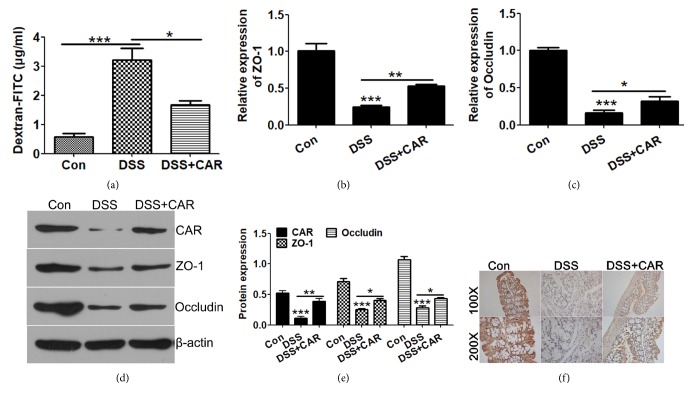
*CAR protects mice from DSS induced colon permeabilization in vivo.* (a) FITC-Dextran permeability assay measurement of colon permeability after different treatment. (b & c) Expression of tight junction gene ZO-1 (a) and Occludin (b) were determined by qPCR. Colon tissue samples were collected after animals were treated with control or DSS/DSS+CAR, mRNA was isolated, and genes were detected by specific primers. (d) Protein levels of CAR and tight junction gene ZO-1 and Occludin were determined by Western blot using specific antibodies, and *β*-actin levels were used as endogenous loading control. (e) Quantitative analysis of CAR, ZO-1, and Occludin protein levels (calibrated with *β*-actin levels of each sample) in (d). (f) Immunohistochemistry staining detection of ZO-1 levels in the colon tissues of different treatment groups. ^*∗*^P < 0.05, ^*∗∗*^P < 0.01, and ^*∗∗∗*^P < 0.001; data represent mean ± s.e.m. from 3 independent experiments with sample size = 3 for each group.

Colon permeation is one of the major factors during the progression of IBD, and in vivo FITC-Dextran assay suggested that overexpression of CAR significantly prevented DSS induced upregulation gut permeability (P<0.05; Figure 7(a)).

Disruption of tight junction proteins has been considered critical factor during IBD pathogenesis, and we analyzed the mRNA abundance of ZO-1 and Occludin. RT-PCR analysis showed that DSS treatment decreased the level of ZO-1, Occludin, and CAR, while CAR overexpression reversed this effect (P<0.05; Figures 7(b) and 7(c)). Consistently, we also observed recovery of ZO-1 and Occludin protein upon CAR overexpression by Western blot (P<0.05; Figures 7(d) and 7(e)). Further immunohistochemistry staining of ZO-1 from colon samples demonstrated increased ZO-1 level after CAR overexpression in DSS induced IBD model (P<0.05; Figure 7(f)).

## 4. Discussion

Initial inflammation of gut epithelia can disrupt barrier function, and increased permeability in turn promotes chronic inflammation and contributes to IBD development [19]. Increased inflammatory cytokines are found in serum, stools, and bowel mucosa of IBD patients and IBD animal models [20], among which TNF-*α* is a potent inflammatory factor which initiates NF-*κ*B transcription factor activation and upregulates gene expression including cytokines and chemokines [21]. In our study, treatment of colon cells with TNF-*α* significantly enhanced the NF-*κ*B and markedly induced levels of IL-21 and IL-23, while overexpression of CAR suppressed these events. Cytokines are well-recognized players which orchestrate the development, recurrence, and exacerbation of inflammation in IBD in time- and space-dependent manner [22, 23]. For example, IL-21 is reported to be upregulated in both CD and UC [24, 25] and activates metalloproteinase 1 production to regulate mucosal ulceration as well as matrix turnover [26]. We also observed suppression of cytokine IL-23 by CAR, which has been reported to be essential for the manifestation of intestinal inflammation via promoting IL-17 and IL-6 [27]. CAR is emerging as an important player in the control of inflammation, we detected decreased CAR expression in TNF-*α* induced colon cell lines, similarly, Zyssy et al. reported that proinflammatory environment is associated with CAR loss in hippocampus [28], and these findings demonstrated that CAR is negatively related to inflammation. Further, we observed downregulation of NF-*κ*B and IL-21/IL-23 expression after CAR overexpression in a ulcerative colitis model. NF-*κ*B is recognized as key transcriptional regulator in the proinflammatory immunological setting of IBD [29]. Activation of NF-*κ*B regulates the secretion of TNF-*α*, IL-1, IL-6, IL-12, and IL-23 in a broad panel of cell-specific ways, playing a central role in the development, maintenance, and chronification of IBD [30, 31]. Suppression of NF-*κ*B by CAR strongly suggests that CAR plays anti-inflammatory role in the pathogenesis of IBD.

The role of CAR in regulating permeability has been suggested in previous studies [15, 32]. Tight junctions are major structures for maintaining intestinal barrier and substance permeability [33]. Damaged TJ can lead to permeation of luminal proinflammatory molecule and induce immune response, contributing to sustained inflammation and tissue damage in IBD pathogenesis [34, 35]. CAR is considered a component of TJ [36], and its colocalization with TJ protein ZO-1 has been demonstrated by coprecipitation and electron microscopy. Disruption of CAR expression affects TJ formation, while transfection of CAR blocks transepithelial passage of ions and large molecules [37]. In both TNF-*α* induced cell and DSS treated mice, we detected significantly protective effect of CAR on maintaining gut barrier function and limit epithelial permeability. At the same time, CAR stabilized the protein levels of ZO-1 and associated transmembrane protein Occludin, suggesting the protective role of CAR in IBD may attribute to its effect on maintaining tight junction protein components and structure.

In summary, the present study has demonstrated a protective role of CAR in the pathogenesis of IBD. Although CAR has become a well-recognized key mediator of inflammation and cell-cell adhesion [15], the precise mechanistic pathways that mediate its functions remain elusive. Intracellular adaptor protein, cytoskeletal remodeling, and other signaling pathways may be involved in the CAR regulation of junction dynamics, and further study to explore how CAR participates in the inflammation response would provide more information for its diagnosis and therapeutic applications.

## 5. Conclusion

In conclusion, our study showed CAR is an important protective factor in the development of IBD. CAR may be used as diagnosis marker and future drug target for treatment of IBD. Additional efforts are required to explore the mechanisms of the anti-inflammatory effect of CAR, as well as how CAR helps maintain functional TJ. Further studies in large clinical cohort of patients are necessary to establish the diagnostic and therapeutic application of CAR in the treatment of IBD.

## Data Availability

The data used to support the findings of this study are available from the corresponding author upon request.

## Abbreviations

CAR: Coxsackie-adenovirus receptor
CD: Crohn's disease
DAI: Disease activity index
DSS: Dextran sulfate sodium
GI: Gastrointestinal
IBD: Inflammatory bowel disease
NF-*κ*B: Nuclear transcription factor kappa B
TJ: Tight junction
TNF-*α*: Tumor necrosis factor-alpha
UC: Ulcerative Colitis
ZO: Zona occludens.

## Additional Points

*Summary*. CAR prevents development of IBD through upregulating tight junction expression and inhibiting inflammation.

## References

[1] Aleksandrova K., Romero-Mosquera B., Hernandez V. (2017). Diet, gut microbiome and epigenetics: emerging links with inflammatory bowel diseases and prospects for management and prevention. *Nutrients*.

[2] Heikenen J. B., Werlin S. L., Brown C. W., Balint J. P. (1999). Presenting symptoms and diagnostic lag in children with inflammatory bowel disease. *Inflammatory Bowel Diseases*.

[3] Kordjazy N., Haj-Mirzaian A., Haj-Mirzaian A. (2017). Role of toll-like receptors in inflammatory bowel disease. *Pharmacological Research*.

[4] Mazal J. (2014). Crohn disease: pathophysiology, diagnosis, and treatment. *Radiologic Technology*.

[5] Ananthakrishnan A. N., Bernstein C. N., Iliopoulos D. (2018). Environmental triggers in IBD: a review of progress and evidence. *Nature Reviews Gastroenterology & Hepatology*.

[6] Nishida A., Inoue R., Inatomi O., Bamba S., Naito Y., Andoh A. (2017). Gut microbiota in the pathogenesis of inflammatory bowel disease. *Clinical Journal of Gastroenterology*.

[7] Ueno A., Jeffery L., Kobayashi T., Hibi T., Ghosh S., Jijon H. (2017). Th17 plasticity and its relevance to inflammatory bowel disease. *Journal of Autoimmunity*.

[8] Geremia A., Arancibia-Cárcamo C. V. (2017). Innate Lymphoid Cells in Intestinal Inflammation. *Frontiers in Immunology*.

[9] Lee S. H. (2015). Intestinal permeability regulation by tight junction: implication on inflammatory bowel diseases. *Intestinal Research*.

[10] Jiang Y., Lin O., Sinha S. R. (2017). Use of tumor necrosis factor alpha inhibitors for inflammatory bowel disease patients with concurrent heart failure. *Digestive Diseases and Sciences*.

[11] Yarur A. J., Kubiliun M. J., Czul F. (2015). Concentrations of 6-thioguanine nucleotide correlate with trough levels of infliximab in patients with inflammatory bowel disease on combination therapy. *Clinical Gastroenterology and Hepatology*.

[12] Kothari M. M., Nguyen D. L., Parekh N. K. (2017). Strategies for overcoming anti-tumor necrosis factor drug antibodies in inflammatory bowel disease: case series and review of literature. *World Journal of Gastrointestinal Pharmacology and Therapeutics*.

[13] Ren W., Yin J., Wu M. (2014). Serum amino acids profile and the beneficial effects of L-arginine or L-glutamine supplementation in dextran sulfate sodium colitis. *PLoS ONE*.

[14] Landy J., Ronde E., English N. (2016). Tight junctions in inflammatory bowel diseases and inflammatory bowel disease associated colorectal cancer. *World Journal of Gastroenterology*.

[15] Raschperger E., Thyberg J., Pettersson S., Philipson L., Fuxe J., Pettersson R. F. (2006). The coxsackie- and adenovirus receptor (CAR) is an in vivo marker for epithelial tight junctions, with a potential role in regulating permeability and tissue homeostasis. *Experimental Cell Research*.

[16] Coyne C. B., Bergelson J. M. (2005). CAR: A virus receptor within the tight junction. *Advanced Drug Delivery Reviews*.

[17] Ortiz-Zapater E., Santis G., Parsons M. (2017). CAR: A key regulator of adhesion and inflammation. *The International Journal of Biochemistry & Cell Biology*.

[18] Srinivasan B., Kolli A. R., Esch M. B., Abaci H. E., Shuler M. L., Hickman J. J. (2015). TEER measurement techniques for in vitro barrier model systems. *Journal of Laboratory Automation*.

[19] Steinbach E. C., Plevy S. E. (2014). The role of macrophages and dendritic cells in the initiation of inflammation in IBD. *Inflammatory Bowel Diseases*.

[20] Nieminen U., Jussila A., Nordling S., Mustonen H., Färkkilä M. A. (2014). Inflammation and disease duration have a cumulative effect on the risk of dysplasia and carcinoma in IBD: a case-control observational study based on registry data. *International Journal of Cancer*.

[21] Mashukova A., Wald F. A., Salas P. J. (2011). Tumor necrosis factor alpha and inflammation disrupt the polarity complex in intestinal epithelial cells by a posttranslational mechanism. *Molecular and Cellular Biology*.

[22] Giuffrida P., Corazza G. R., Di Sabatino A. (2018). Old and new lymphocyte players in inflammatory bowel disease. *Digestive Diseases and Sciences*.

[23] Norouzinia M., Chaleshi V., Alizadeh A. H. M., Zali M. R. (2017). Biomarkers in inflammatory bowel diseases: insight into diagnosis, prognosis and treatment. *Gastroenterology and Hepatology from Bed to Bench*.

[24] Fina D., Caruso R., Pallone F., Monteleone G. (2007). Interleukin-21 (IL-21) controls inflammatory pathways in the gut. *Endocrine, Metabolic & Immune Disorders—Drug Targets*.

[25] Ebrahimpour S., Shahbazi M., Khalili A. (2017). Elevated levels of IL-2 and IL-21 produced by CD4+ T cells in inflammatory bowel disease. *Journal of Biological Regulators and Homeostatic Agents*.

[26] Ravi A., Garg P., Sitaraman S. V. (2007). Matrix metalloproteinases in inflammatory bowel disease: Boon or a bane?. *Inflammatory Bowel Diseases*.

[27] Katsanos K. H., Papadakis K. A. (2017). Inflammatory bowel disease: updates on molecular targets for biologics. *Gut and Liver*.

[28] Zussy C., Loustalot F., Junyent F. (2016). Coxsackievirus adenovirus receptor loss impairs adult neurogenesis, synapse content, and hippocampus plasticity. *The Journal of Neuroscience*.

[29] Neurath M. F., Pettersson S., Zum Buschenfelde K.-H. M., Strober W. (1996). Local administration of antisense phosphorothioate oligonucleotides to the p65 subunit of NF-*κ*B abrogates established experimental colitis in mice. *Nature Medicine*.

[30] Becker C., Wirtz S., Blessing M. (2003). Constitutive p40 promoter activation and IL-23 production in the terminal ileum mediated by dendritic cells. *The Journal of Clinical Investigation*.

[31] Becker C., Wirtz S., Ma X., Blessing M., Galle P. R., Neurath M. F. (2001). Regulation of IL-12 p40 promoter activity in primary human monocytes: roles of NF-*κ*B, CCAAT/enhancer-binding protein *β*, and PU.1 and identification of a novel repressor element (GA-12) that responds to IL-4 and prostaglandin E21. *The Journal of Immunology*.

[32] Oh Y. S., Nah W. H., Choi B., Kim S. H., Gye M. C. (2016). Coxsackievirus and adenovirus receptor, a tight junction protein, in peri-implantation mouse embryos. *Biology of Reproduction*.

[33] Coskun M. (2014). Intestinal epithelium in inflammatory bowel disease. *Frontiers in Medicine*.

[34] Fries W., Belvedere A., Vetrano S. (2013). Sealing the broken barrier in IBD: intestinal permeability, epithelial cells and junctions.. *Current Drug Targets*.

[35] Watson A. J. M., Hughes K. R. (2012). TNF-*α*-induced intestinal epithelial cell shedding: implications for intestinal barrier function. *Annals of the New York Academy of Sciences*.

[36] Cohen C. J., Shieh J. T. C., Pickles R. J., Okegawa T., Hsieh J.-T., Bergelson J. M. (2001). The coxsackievirus and adenovirus receptor is a transmembrane component of the tight junction. *Proceedings of the National Acadamy of Sciences of the United States of America*.

[37] Caruso L., Yuen S., Smith J., Husain M., Opavsky M. A. (2010). Cardiomyocyte-targeted overexpression of the coxsackie-adenovirus receptor causes a cardiomyopathy in association with *β*-catenin signaling. *Journal of Molecular and Cellular Cardiology*.

